# Automated Segmentation of Breast Cancer Focal Lesions on Ultrasound Images

**DOI:** 10.3390/s25051593

**Published:** 2025-03-05

**Authors:** Dmitry Pasynkov, Ivan Egoshin, Alexey Kolchev, Ivan Kliouchkin, Olga Pasynkova, Zahraa Saad, Anis Daou, Esam Mohamed Abuzenar

**Affiliations:** 1 Medical Institute, Department of Radiology and Oncology, Mari State University, Ministry of Education and Science of Russian Federation, 1 Lenin Square, Yoshkar-Ola 424000, Russia; jungl91@mail.ru (I.E.); o.o.pasynkova@yandex.ru (O.P.); zahraaalisaad2@gmail.com (Z.S.); hamzamo565@gmail.com (E.M.A.); 2 Kazan State Medical Academy—Branch Campus of the Federal State Budgetary Educational Institution of Further Professional Education, Russian Medical Academy of Continuous Professional Education, Ministry of Healthcare of the Russian Federation, 36 Butlerov St., Kazan 420012, Russia; 3 Institute of Computational Mathematics and Information Technologies, Kazan Federal University, 18 Kremlevskaya St., Kazan 420008, Russia; kolchevaa@mail.ru; 4 Pediatric Faculty, Kazan Medical University, Ministry of Health of Russian Federation, 49 Butlerov St., Kazan 420012, Russia; hirurgivan@mail.ru; 5 Pharmaceutical Sciences Department, College of Pharmacy, QU Health, Qatar University, Doha 2713, Qatar

**Keywords:** ultrasound image, lesion, breast cancer, random forest classifier, region of interest, segmentation

## Abstract

Ultrasound (US) remains the main modality for the differential diagnosis of changes revealed by mammography. However, the US images themselves are subject to various types of noise and artifacts from reflections, which can worsen the quality of their analysis. Deep learning methods have a number of disadvantages, including the often insufficient substantiation of the model, and the complexity of collecting a representative training database. Therefore, it is necessary to develop effective algorithms for the segmentation, classification, and analysis of US images. The aim of the work is to develop a method for the automated detection of pathological lesions in breast US images and their segmentation. A method is proposed that includes two stages of video image processing: (1) searching for a region of interest using a random forest classifier, which classifies normal tissues, (2) selecting the contour of the lesion based on the difference in brightness of image pixels. The test set included 52 ultrasound videos which contained histologically proven suspicious lesions. The average frequency of lesion detection per frame was 91.89%, and the average accuracy of contour selection according to the IoU metric was 0.871. The proposed method can be used to segment a suspicious lesion.

## 1. Introduction

Breast cancer (BC) is a commonly encountered malignancy characterized by a high degree of aggressiveness. Despite significant achievements in the diagnosis and treatment of BC, this issue remains relevant today. In 2020, breast cancer ranked first in the global incidence rate among both genders (11.7% of all malignancies) and fifth in the structure of overall oncological mortality (6.9%). Among women, this pathology is the most frequently diagnosed malignancy (24.5%) and ranked second in terms of mortality (15.5%) [1,2].

Although mammography remains the primary method for the early detection of BC, it often fails to establish an accurate diagnosis, necessitating the use of other methods for further differentiation of identified changes. The main method for non-invasive differential diagnosis of BC is ultrasound (US), which, despite its undeniable and well-known advantages, has several serious limitations, the most significant of which is operator dependence partially due to the variability of human visual perception [3].

This underscores the relevance and necessity of developing approaches for the automated analysis of US images to identify changes corresponding to BC and to subsequently automate their differentiation, for example, by using previously developed approaches by the authors [4,5].

Traditional image segmentation approaches, such as threshold-based methods, boundary analysis, and region growing, complicate the achievement of optimal segmentation results in real time. In response to these challenges, researchers are actively modifying and supplementing existing algorithms. For example, Liu et al. [6] developed a new structure for the fully automated segmentation of lesions in breast ultrasound images, applying morphological filtering and adaptive thresholding using the Otsu method to identify the region of interest (ROI) and the shape of the lesion. Wu et al. [7] focused on evaluating the quality of ultrasound images using deep convolutional networks, demonstrating the effectiveness of their approach in experimental results. Following the initialization of contours, Liu et al. [6] improved the Chan–Vese model for achieving more accurate segmentation.

Wang and Jiao [8] applied a Simplified Pulse-Coupled Neural Network, complemented by morphological processing and fuzzy mutual information, to extract the tumor area from binary images, using the highest fuzzy mutual information as the selection criterion. Chen et al. [9] proposed a method for a cascade convolutional neural network (CNN) called C-Net, which consists of several networks. This model includes three networks and is trained using deep supervision. The first network generates saliency maps, which are then processed using an attention network and a refinement network, passing the segmentation result to the output.

Despite significant advances in deep learning for processing natural images, breast US images still face challenges such as low resolution, weak contrast, and high noise levels. Although modern deep learning methods appear promising, they have their drawbacks, such as insufficient model justification and difficulties in creating representative training datasets. Therefore, it is essential to develop effective algorithms for the segmentation, classification, and analysis of US images to ensure accuracy and reliability in diagnosis.

In this context, the aim of this work was to develop a methodology for the automated detection of pathological lesions in breast ultrasound images and their segmentation, as well as to test this methodology on clinical materials.

## 2. Materials and Methods

### 2.1. Materials

**Figure 1:**
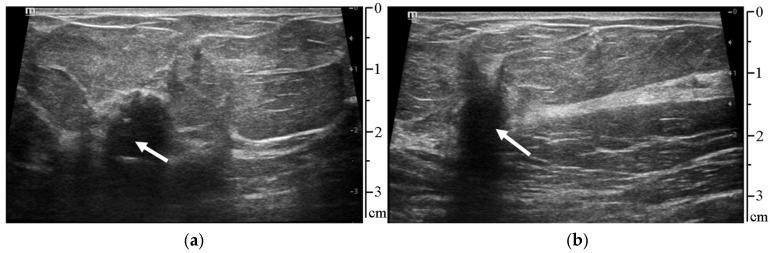
Frames from two different ultrasound video sequences; the arrows indicate (**a**) histologically proven mucinous breast cancer; and (**b**) histologically proven ductal breast cancer.

The initial data for developing and testing the proposed method were the US video sequences with a duration of 5 s, a resolution of 1200 × 900 pixels, and a frequency of 12 frames per second. Each frame of the video sequence was a digitized 8-bit US image in grayscale, which contained histologically proven breast carcinoma lesions (see Figure 1). The Mindray DC8-EX medical US system (Shenzhen Mindray Bio-Medical Electronics Co., Ltd., Shenzhen, China) was used to obtain the video sequences.

For the task of classifying normal tissue during the training of the random forest model, 55 ultrasound images of the breast were used, obtained from 55 different patients. Images containing suspicious pathological lesions were not used. A total of 100 different frames were used to test the classification method (analyzing correctly classified pixels of various tissue types), obtained from 100 different ultrasound video sequences of different patients, which were not used in the training process of the random forest model. Images from different patients were used for model training and subsequent testing in the study. There is no overlap between the patient cases used for the training and testing sets. But there is an overlap between patient cases in the first (tissue classification) and second (contour segmentation) stages, which are used only for method testing. To evaluate the accuracy of classification, the average percentage of correctly classified pixels for all 100 frames was calculated.

To assess the quality of the contour segmentation of the lesion and to test the combined method, 52 video sequences from 52 different patients with various types of pathological changes were used. All data was obtained in Clinical Oncology Dispensary of Mari El Republic, Yoshkar-Ola, Russia. The assessment methodology is presented in Section 2.2.4.

### 2.2. Methods

#### 2.2.1. General Description of the Methodology

The developed method involves frame-by-frame processing of the video sequence and includes two stages of processing US images. In the first stage, normal tissue (background) classification is performed, the suppression of which highlights the region of interest (ROI) in the image containing the suspicious lesion and determines the approximate center of the lesion itself. When developing the first stage, the authors assumed that by classifying normal breast tissues in the image, in the presence of a pathological lesion, there would remain image pixels that would either not be classified as normal tissues or would be classified as such with low probability. That is, the task of determining the ROI containing the lesion can be considered as an inverse problem. The implementation of this stage is based on the classification of normal breast tissues in the image, for which a machine learning methodology was applied—a random forest classifier with the Gini criterion [10].

**Figure 2:**
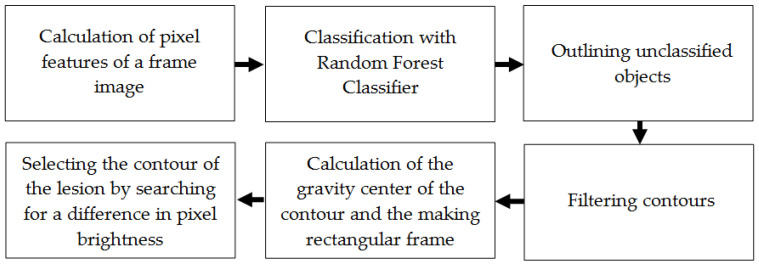
Block diagram of the proposed method for identifying the contour of a lesion in ultrasound video frames.

In the second stage, the actual contour extraction of the lesion is performed. Figure 2 presents a flowchart of the proposed method for extracting the contour of the lesion in the frames of the ultrasound video sequence.

#### 2.2.2. Classification of Normal Tissues

To address this task, we used a random forest classifier, which is an ensemble learning model that utilizes multiple decision trees for prediction. During the training process, the algorithm forms a multitude of trees by randomly selecting both data and features. Each tree is trained on a separate subset of data and makes decisions based on a random set of features. After all trees have been trained, the final prediction is made by combining the results obtained from each individual tree [11].

**Figure 3:**
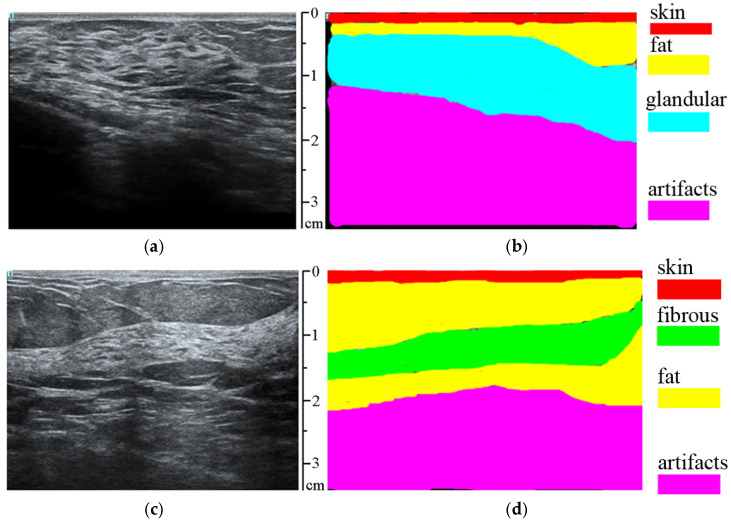
(**a**,**c**) Original ultrasound images; (**b**,**d**) ultrasound images with marked tissues on them (skin, fat, fibrous tissue, glandular tissue, and artifacts).

Fifty-five ultrasound images with annotated tissue types, as specified by a specialist, were used: skin, fat, fibrous tissue, fibrous bands, glandular tissue, and artifacts for training the classifier (see Figure 3). Images containing suspicious pathological lesions were not used in this step. Cross-validation was performed with data that were not involved in the training of the random forest model.

The set of classifying features consisted of local structures around each pixel (eigenvalues of the Hessian matrix), which were computed at different scales of Gaussian smoothing. Let the ultrasound image be represented as a two-dimensional matrix *F* (*f*) of size (*n* × *m*), where *f* is the pixel intensity. Then, Gaussian smoothing is performed with a specified parameter σ for each element of the matrix *f* (*x*, *y*):

(1) $$F^{*}(f^{*})=g(x,y)\times f(x,y),g(x,y)=\frac{1}{2\pi \sigma^{2}}e^{-\frac{x^{2}+y^{2}}{2\sigma^{2}}}$$

where *x*, *y* are the coordinates of the point, and σ is the standard deviation of the normal distribution. σ is usually equal to σ_min_, 2σ_min_, 4σ_min_, …, (2*n* − 1)σ_min_, where (2*n* − 1)σ_min_ ≤ σ_max_ = 128. After applying the Gaussian filter, the Hessian matrix is calculated for each pixel value:

(2) $$H(x)=\left(\begin{array}{ccc} h_{11} & \ldots & h_{1k}\\ \ldots & \ldots & \ldots \\ h_{k1} & \ldots & h_{kk} \end{array}\right)$$

where $h_{ij}=\frac{\partial^{2}F^{*}(x)}{\partial x_{i}\partial x_{j}}$, *i*, *j* = 1, …, *n*.

The first and second eigenvalues of the Hessian matrix λ*_i_* are taken as training features:

(3) $$\left|H(x)-\lambda E\right|=\left(\begin{array}{ccc} h_{11}-\lambda & \ldots & h_{1k}\\ \ldots & \ldots & \ldots \\ h_{k1} & \ldots & h_{kk}-\lambda \end{array}\right)=0.$$

So, we obtain a set of 16 features. The local binary pattern (LBP) descriptor is used as an additional feature, which encodes the pixels of the image by comparing the central pixel with its neighbors, and the result is considered a binary pattern number. When computing LBP, the difference between the current pixel and its neighbors in a 3 × 3 aperture is taken. This is defined by the following equation:

(4) $$\mathrm{LBP}=\sum_{p=0}^{P-1}S(g_{p}-g_{c})2^{P},\;S(x)=\left\{\begin{array}{c} 1,x\geq 0\\ 0,x<0 \end{array}\right.,$$

where *g_p_* is the neighbor’s value, *g_c_* is the central pixel’s value, and *P* is the number of neighbors. If the central pixel matches or is less than the neighboring pixel, it is marked as 1; otherwise, it is marked as 0.

As a result, the obtained set of 17 features is fed into a random forest classifier with the Gini criterion, where the final classifier is expressed by the equation:

(5) $$a(x)=\frac{1}{N}\sum_{i=1}^{N}b_{i}(x),$$

where *N* is the number of trees (we chose *N* as 50), *b* is the decision tree, and *x* is the set generated from the data. A majority voting solution is chosen for the classification task.

We selected 50 trees after using the cross-validation method, where the number of trees was gradually increased (20, 30, …, 100), and the model quality was assessed on validation data. At 50 trees, the classification quality significantly ceased to improve (reaching a “plateau” point). It is important to consider that increasing the number of trees can improve performance but may also lead to overfitting.

The random forest model provides class probabilities based on the proportion of trees that voted for each class. This allows the random forest to be used not only for classification but also for tasks where the model’s predictive confidence is important.

In a random forest, each tree in the ensemble “votes” for the class to which it believes the object (in our case, the pixel) belongs. For a multi-class classification task, each tree predicts one class. The number of votes for each class is counted after all the trees have voted. For example, if 50 trees are used and 30 of them voted for the Fat tissue class while 20 voted for the Skin tissue class, the proportion of votes for the Fat tissue class would be 60%, and it would be 40% for the Skin tissue class. These vote proportions are interpreted as probabilities of the object’s membership in each class. So, the probability of assigning the object to the Fat tissue class will be 0.6, and to the Skin tissue class will be 0.4.

Therefore, in the case where the probability of assigning a pixel to one of the classes (skin, fat, fibrous tissue, fibrous bands, glandular tissue, and artifacts) is less than 20%, this pixel is not annotated and is considered potentially related to the lesion.

**Figure 4:**
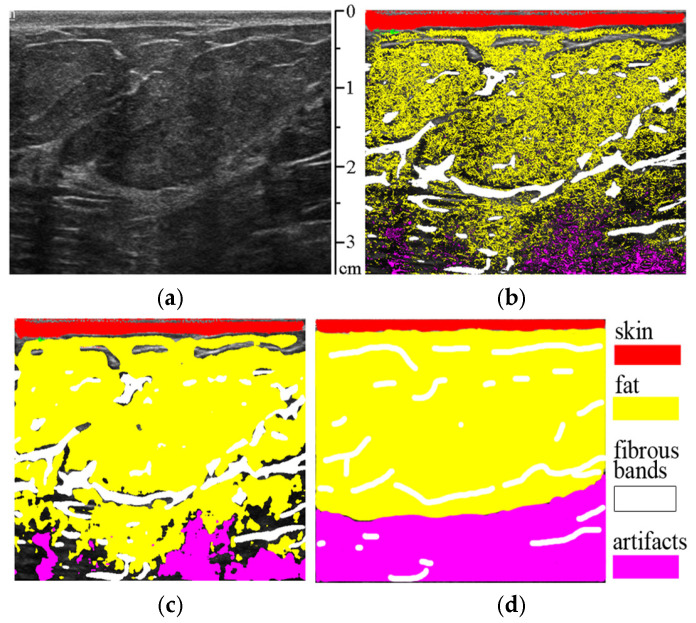
(**a**) Original ultrasound image; (**b**) result of tissue classification by the random forest classifier; (**c**) after applying the morphological dilation operation and median filter; (**d**) ground truth.

The classification result of tissues in ultrasound images using the trained random forest model is shown in Figure 4b.

From Figure 4b, it can be seen that after automatic tissue classification, there are a few unmarked pixels that were not classified into one of the groups due to having a low probability of belonging. Therefore, to avoid this drawback, the morphological dilation operation is applied to merge the unmarked pixels, along with a median filter to eliminate false “outliers”—incorrectly marked points (see Figure 4c). These filters are applied to the obtained classified image masks.

Then, all unmarked objects in the image are identified, and those that are artifacts—typically elongated with an average pixel intensity > 93, eccentricity > 0.9, and a ratio of the object’s contour length (perimeter) to its area > 0.1—are removed:

(6) $$I_{\mathrm{aver}}=\frac{1}{N}\sum_{i=1}^{N}I_{i}>93;\;e=\sqrt{1-\frac{b^{2}}{a^{2}}}>0.9;\;R=\frac{P}{S}>0.1;$$

where *I* is the pixel intensity, *N* is the number of pixels in the object, *a* and *b* are the major and minor axes, respectively, *P* is the perimeter of the object, and *S* is the area of the object (or the number of pixels in the object).

To find the centers *A*(*x*_0_, *y*_0_) of all remaining objects, the center of mass of their contours is calculated:

(7) $$x_{0}=\frac{1}{N}\sum_{i=1}^{N}x_{i},\;y_{0}=\frac{1}{N}\sum_{i=1}^{N}y_{i}$$

where *x*, *y* are the coordinates of the contour pixels, and *N* is the number of contour pixels.

#### 2.2.3. Segmentation of the Pathologic Lesion

**Figure 5:**
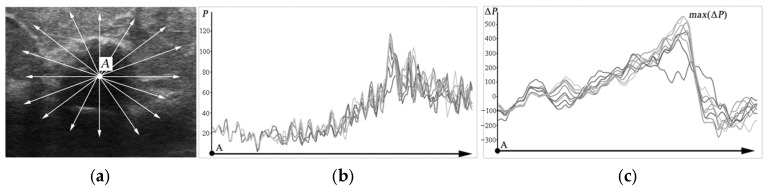
(**a**) Rays drawn from the center of lesion *A*; (**b**) brightness values of the pixels *P_i_* lying on the rays; (**c**) graphs of the brightness gradients Δ*P_i_* of the pixels lying on the rays.

To implement the second stage of the proposed method, all classified tissues are removed from the original image, and all remaining areas are highlighted. Then, to extract the precise contour of the pathological lesion, the features of the brightness gradient of the image pixels are taken into account. For this, rays are drawn in 360 degrees from the center of the lesion found in the previous stage (point *A*) along the entire circumference of the lesion (see Figure 5a). The rays are drawn at intervals of 0.5 degrees. Thus, a total of *T* = 720 rays are drawn.

The brightness gradient of the pixels located on the rays (see Figure 5b) is calculated using a one-dimensional sliding window of a specified size (Figure 5c):

(8) $$\Delta P_{i}=\sum_{j=1}^{S}P_{i+j}-\sum_{j=1}^{S}P_{i-j},\;i=S,\;\ldots ,\;N-S$$

where *N* is the number of pixels on the ray, *P_i_* = *P*(*x_i_*, *y_i_*) is the brightness of the pixel corresponding to the coordinates *x_i_* and *y_i_* on the ray, and *S* is the size of the sliding window.

**Figure 6:**
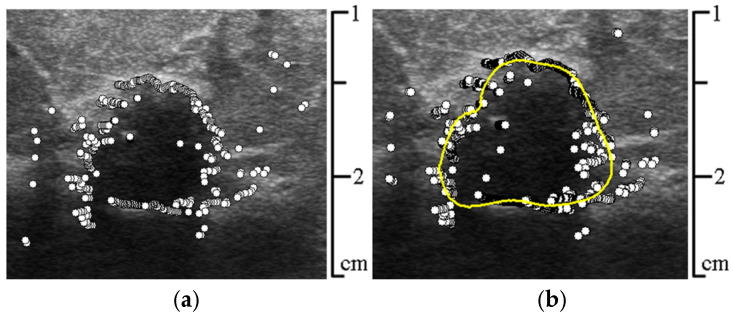
(**a**) Points corresponding to the extrema of the brightness gradient on the rays; (**b**) constructed averaged cubic regression for them (solid thick yellow line).

The largest *max*(Δ*P_i_*) brightness gradients (extrema) corresponded to the approximate boundaries of the area necessary for contouring. By transferring the coordinates of the extrema of the brightness gradient to the original image, we obtain the approximate boundaries (see Figure 6a).

Next, the obtained boundaries of the area undergo subsequent correction by filtering their points—if the Euclidean distance between the boundary points of the area and the points of their regression line lying on the same ray exceeds a threshold, then such a point is replaced by an interpolated one. The threshold is calculated using the Niblack method [12].

To implement this approach, the entire array of *T* = 720 points (*x_j_*, *y_j_*) of the boundary of the lesion is divided into *U* subarrays of size *M* = 180 points using a sliding window method of size *W* = 10 points. To ensure the closure of the contour, the first *M*_0_ = 180 points of the array are appended to the end of the array of *T* points before its division: T* = *T* + *M*_0_. Then, the following method is applied to each subarray: Let *X* = *x*_1_, *x*_2_, …, *x_m_* be the subarray of the *x* coordinates of the contour points of the lesion, and *Y* = *y*_1_, *y*_2_, …, *y_m_* be the subarray of the *y* coordinates of the contour points of the lesion, where m∈M; then, *R* = *r*_1_, *r*_2_, …, *r_m_* and *Φ* = φ_1_, φ_2_, …, φ*_m_* are their representations in polar coordinates, where $r_{j}=\sqrt{{\left(x_{A}-x_{j}\right)}^{2}+{\left(y_{A}-y_{j}\right)}^{2}}$, φ*_j_* = *arctg* (*y_j_*/*x_j_*), *j* = 1, …, *M*, and *x_A_*, *y_A_* are the coordinates of the center of the lesion (point *A*) highlighted in the initial stage of processing.

The equation of cubic regression for the polar coordinate system takes the form:

(9) $${\bar{r}}_{j}=a_{3}\phi_{j}^{3}+a_{2}\phi_{j}^{2}+a_{1}\phi_{j}+a_{0},$$

where *a*_0_, *a*_1_, *a*_2_, and *a*_3_ are the coefficients of the cubic regression.

Since this approach is applied with a sliding window *W* across the entire augmented array T*, this reduces the deviations (errors) in the values of the cubic regression function ${\bar{r}}_{j}$ corresponding to the beginning and end of the subarray *U*, due to the averaging of the obtained values—${\bar{r}}_{j}^{\mathrm{avr}}$. In Figure 6b, the thick solid yellow line shows the averaged cubic regression for the extrema of the brightness gradient points.

Thus, for each point of the approximate boundary, we obtain a pair of values *r_j_* and ${\bar{r}}_{j}^{\mathrm{avr}}$, for which we calculate $\Delta r_{j}=\left|{\bar{r}}_{j}^{\mathrm{avr}}-r_{j}\right|$. To correct the points, we find the threshold using the Niblack method:

*B* = *m* + *k* × *s* (10)

where *m* and *s* are the mean and standard deviation of Δ*r_i_*, respectively, with the coefficient *k* = 0.2.

If Δ*r_j_* exceeds the calculated threshold *B*, then the corresponding coordinates of the point (*x_j_*, *y_j_*) are replaced with interpolated ones. This approach allowed us to eliminate sharp changes in the object’s boundaries, which would yield false results in the area extraction.

#### 2.2.4. Quality Assessment of Lesion Segmentation

Frames from ultrasound video sequences containing morphologically verified tumors (52 frames from 52 videos, one frame per video) were used to assess the quality of lesion contour segmentation, annotated by a specialist physician. The outlined contours of the lesion itself were used as the ground truth. Due to the characteristics of ultrasound images, which typically have various artifacts, as well as low contrast from weak ultrasound signals that are absorbed by the overlying tissues, only the upper hemisphere of the lesion was considered for contour accuracy assessment, and the following metrics were calculated:

1. The mean value $\bar{d}$ and standard deviation σ of the Euclidean distance between the nearest points of the segmented contour and the ground truth (in pixels):
  (11) $$\bar{d}=\frac{\sum_{N}d_{i}}{N},\;\sigma =\sqrt[{}]{\frac{\sum_{N}{\left(d_{i}-\bar{d}\right)}^{2}}{N}}$$
  where *d_i_* is the Euclidean distance between the nearest contour points, and *N* is the number of contour points.
2. The ratio of the area of intersection to the union of the segmented contour and the ground truth:
  (12) $$IoU=\frac{A\cap B}{A\cup B},$$
  where *A* is the area of the lesion segmented by the proposed method, and *B* is the area of the ground truth (in pixels).
3. Precision, Recall, and *F*1-*score*:
  (13) $$\mathrm{Precision}=\frac{\mathrm{TP}}{\mathrm{TP}+\mathrm{FP}},\;\mathrm{Recall}=\frac{\mathrm{TP}}{\mathrm{TP}+\mathrm{FN}},\;F1\text{-}\mathrm{score}=\frac{2\cdot \mathrm{Precision}\cdot \mathrm{Recall}}{\mathrm{Precision}+\mathrm{Recall}},$$
  where $\mathrm{TP}=\frac{A\cap B}{B}$, $\mathrm{FP}=\frac{A\cup B-B}{B}$, FN=1−TP.

True Positive (*TP*) is the number of correctly predicted pixels, False Positive (*FP*) is the number of incorrectly predicted pixels, and False Negative (*FN*) is the number of unpredicted pixels of the ground truth.

To reduce the error in manual contour segmentation used as the ground truth, the specialist physician performed the segmentation of the same contour on the same ultrasound frame ten times. Only one frame from each video was used in the evaluation. Based on these repeated segmentation samples, variability was calculated as the mean value $\bar{d}$ and standard deviation σ of the Euclidean distance between the nearest points of the segmented contours.

The manual tracing of the ground truth contour of a lesion by a trained radiologist can vary for several reasons. First, due to the subjectivity of perception, radiologists may interpret ultrasound images differently based on their experience, knowledge, and individual perception, which can lead to discrepancies in defining the boundaries of the lesion. Second, the characteristics of the pathology—some lesions may have ill-defined or unclear boundaries—make accurate delineation difficult. Additionally, errors may occur due to fatigue, inattentiveness, or other factors related to human involvement.

To minimize such errors and determine the uncertainty in tracing the ground truth, contour tracing was performed multiple times by the specialist physician, allowing for the calculation of the average uncertainty in tracing, defined by the mean value $\bar{d}$ and the standard deviation σ. This approach will enable the future evaluation of the difference between the accuracy of contour segmentation using the proposed automated method and the uncertainty in tracing the ground truth by the radiologist.

## 3. Results

### 3.1. Classification and Suppression of Normal Tissues

**Figure 7:**
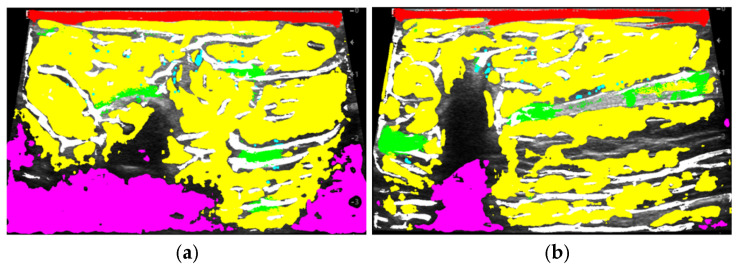

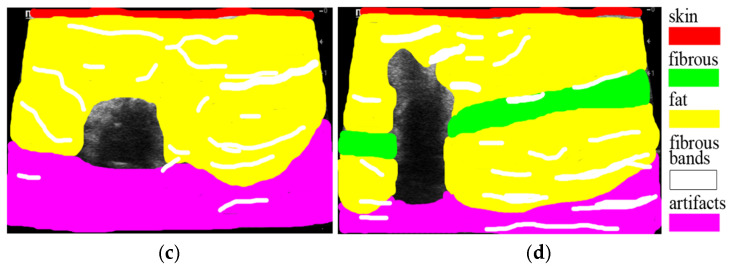
Results of tissue classification on frames from the ultrasound video sequences corresponding to Figure 1. (**a**) Histologically proven mucinous breast carcinoma; (**b**) histologically proven ductal breast carcinoma from different video sequences of another patient; (**c**,**d**) ground truths.

Figure 7 shows the results of applying the proposed method to ultrasound video frames corresponding to Figure 1, containing various suspicious lesions. The textural features of these lesions were not included in the training set of the random forest model.

The analysis of correctly classified pixels of various tissue types was performed on 100 frames of ultrasound video sequences that were not used in the training process of the random forest model, and the average percentage of correctly and incorrectly classified pixels was calculated for all frames. Table 1 presents the results of the accuracy assessment for normal tissue classification.

The proposed method can be used to delineate the ROI containing a suspicious lesion, as seen from Figure 7, and the results are presented in Table 1. To implement this idea, all classified tissues are removed from the original image, and all remaining areas are segmented.

**Figure 8:**
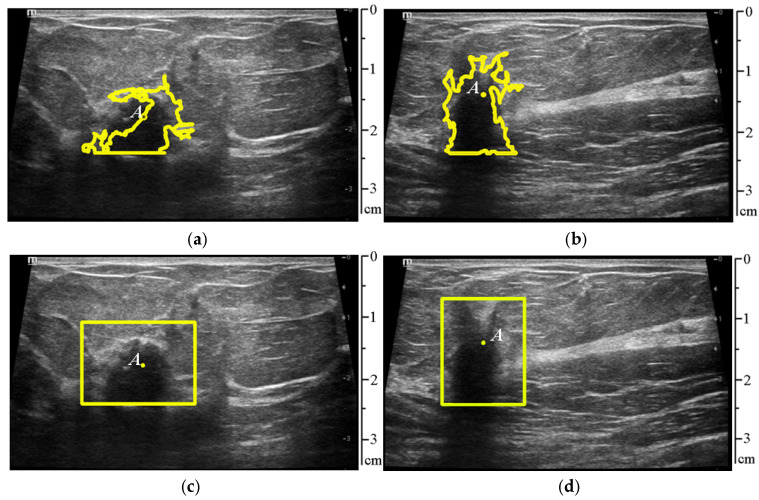
The result of identifying unclassified objects in the frames of ultrasound video sequences corresponding to Figure 1. (**a**,**b**) Show the highlighted objects after additional shape filtering, (**c**,**d**) show the rectangular areas circled around them, which will be the ROI.

Figure 8a,b show the highlighted objects after additional shape filtering. Figure 8c,d display the rectangular areas circled around them, which will be the ROI.

### 3.2. Segmentation of Pathologic Lesion

**Figure 9:**
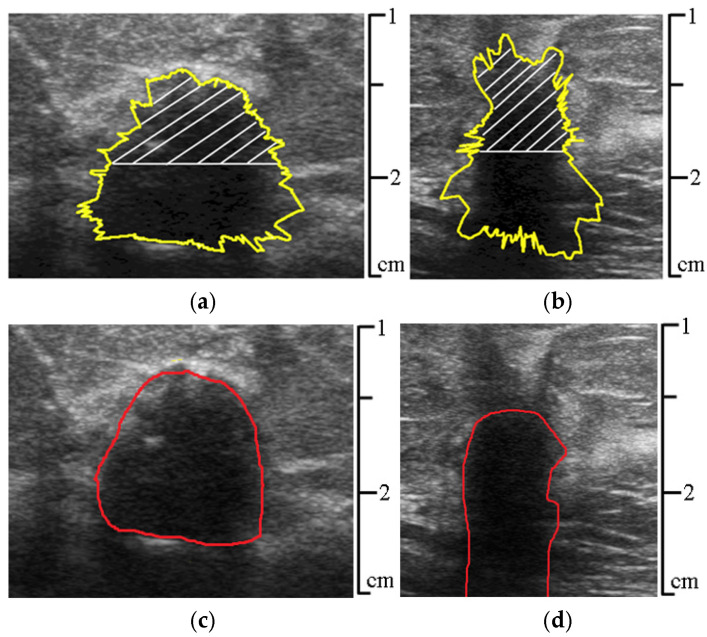
(**a**,**b**) Segmented contours of lesions using pixel intensity gradient detection along rays drawn from the center of gravity of the contour (yellow); (**c**,**d**) contours of the lesion outlined by the specialist physician (red). The images correspond to Figure 1.

Figure 9a,b show the results of the contour segmentation outcomes (yellow line), with the upper hemisphere of the lesion highlighted by white lines. Figure 9c,d show the contours of the lesion outlined by the specialist physician (red line).

Table 2 shows the results of the quality assessment of the proposed contour segmentation method.

Table 3 shows the results of variability assessment for the ground truth contours across fifty-two video sequences and their average evaluation. The first video of the testing series contains the histologically proven mucinous breast carcinoma, and the second shows histologically proven ductal breast carcinoma. The other 50 videos also feature various types of carcinomas in different patients.

From Table 2 and Table 3, it is evident that the mean segmentation errors for the pathological lesion of the breast are $\bar{d}$ = 15.37 pixels across all 52 videos, which, considering the error from the manual segmentation by the specialist (13.73 pixels), is insignificant. The average *IoU* metric is 0.871.

A comparison of the proposed method with previously described ones is given in Table 4. The table shows that the proposed method can compete with other modern methods effectively.

### 3.3. Testing of the Combined Method

**Figure 10:**
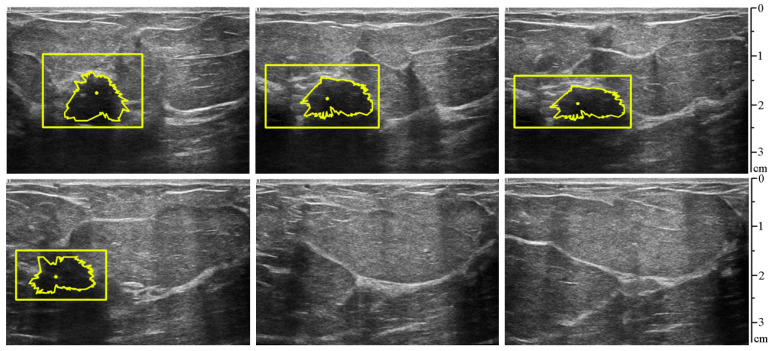
Frame-by-frame processing of the video ((**left**) to (**right**), (**top**) to (**bottom**): frame 1, frame 10, frame 20, frame 30, frame 40, frame 50). In frames 40 and 50, the ROI was not detected due to the absence of a suspicious lesion.

The proposed method was tested on ultrasound video sequences with and without the suspicious lesion. The video duration was 5 s, with a frame rate of 12 frames per second. Figure 10 shows the frame-by-frame processing of the ultrasound video, where the first and second stages of the proposed method were tested, which involves detecting the ROI in the image (rectangular frame), locating the center of the lesion (yellow dot), and directly segmenting the contour of the lesion.

Table 5 shows the results of lesion detection in the frames of fifty-two ultrasound video sequences, where the first video contains histologically proven mucinous breast carcinoma, and the second video contains histologically proven ductal breast cancer.

Despite the possible differences in the textural features of the pathological lesions and the presence of various structures of normal tissue in each individual video, the detection accuracy of the pathological lesions remains high (88.34% for the first type of lesion, 90.00% for the second one, and 91.89% across all 52 videos).

So, the proposed method can be used to delineate the ROI containing a suspicious lesion and for its subsequent segmentation.

## 4. Discussion

The complexity of US image analysis lies in the fact that the images themselves are subject to various types of noise and artifacts from reflections, which can complicate their interpretation and degrade diagnostic quality. Moreover, US waves interact with different tissues according to complex laws, resulting in various effects such as scattering, reflection, and absorption. US images of different tissue types also exhibit high variability depending on their structural characteristics. Additionally, different tissues often have similar acoustic properties, making the task of differentiating them challenging. These effects must be taken into account when processing US images.

Currently promising deep learning methods [19,20,21] have a number of drawbacks, including the fact that the model itself is often insufficiently justified because of the lack of interpretability of the trained model. Even if the algorithm performs well, its results must be interpretable for physicians. Therefore, we need methods that not only provide results but also explain the basis on which they were obtained. The interpretation concept proposed in [22] considers only the shape of the examined lesion. Furthermore, training machine learning algorithms requires a large amount of annotated data. However, in medicine, it is often challenging to obtain a sufficient number of well-annotated ultrasound images, especially for creating a representative training database due to the variability in patient anatomy (which can differ significantly) and the inconsistency of conditions (scanning may be performed on different US systems, with variable settings, etc.). The issue of insufficiently annotated data for ultrasound images is further complicated by the fact that ultrasound images contain specific context and characteristics that may be lost or distorted when applying various augmentation methods (e.g., rotation or scaling). Additionally, ultrasound images may have biological constraints that need to be taken into account during augmentation. Changes that are artificially introduced to the images may not reflect real changes in tissue or anatomy. A potential solution to these problems could be the incorporation of mathematical models representing the examined objects into the architecture of the deep learning model. A similar attempt was made in [23], where a segmentation result with an IoU metric of only 85.634 was achieved.

Therefore, it is necessary to develop effective algorithms for the segmentation, classification, and analysis of US images to ensure diagnostic accuracy and reliability.

We proposed the random forest method for tissue classification and identification of ROI containing pathologic lesions. Its main advantages include its ability to handle multidimensional data, resilience to outliers and noise, as well as its capacity to uncover complex nonlinear relationships between features and the target variable [11].

The relatively high error rate in classifying tissue artifacts is associated with the significant variability of their textural characteristics and low contrast due to weak ultrasound signals, which are absorbed as they pass through all preceding tissues. However, this drawback can be mitigated by additionally using region growing methods [24] or active contours [25], while the proposed classification method can serve as an initial approximation for them.

Additionally, the frequency of detecting lesions in ultrasound video frames can be improved by incorporating the analysis of neighboring frames. The proposed method did not consider such an analysis, focusing solely on individual frames. However, if a lesion is detected in the current frame but not in the subsequent one, an additional filter can be added for a more detailed analysis, or an averaging of pixel values from the two frames can be performed. To reduce the percentage of false detections, an additional classification module for the detected lesions could be included (for example, [5]), which would filter out false positive results.

Areas of significant noise, strong blur, and poor contrast may be characterized by the absence of a strong brightness gradient change at the boundary of the breast lesion, which can indeed complicate the subsequent segmentation of the lesion. To minimize such errors, it is essential to apply adaptive approaches that can adjust parameters based on the assessment of the input image quality. For example, if the noise level is high, the algorithm may modify the gradient weights or the size of the window used for its calculation. Additionally, the methodology proposed in the paper uses averaging of regression lines constructed for the previously identified points of lesions, which also helps to minimize the error.

The choice of classification features, such as the eigenvalues of the Hessian matrix (or the matrix of second derivatives), is justified by the fact that the Hessian matrix is typically applied in the context of the unpredictable shape and structure of objects. The eigenvalues of the Hessian matrix can contain important information about the local features of the image, such as curvature and the shape of objects. Features based on eigenvalues are often more robust to noise compared to other methods, as they extract information based on the global structure of the image [26]. In general, eigenvalues of the Hessian matrix as features for image classification can offer a combination of robustness and informative value. The advantage of using LBP as image features for classification is that they have relatively high robustness to noise, as the results mainly depend on local relationships between neighboring pixels. Additionally, LBP focuses on local textures, making it suitable for analyzing image details and improving the identification of object texture characteristics [27].

It is also important to note that the classification results obtained using LBP features and the eigenvalues of the Hessian matrix, unlike those obtained from deep learning methods, are easier to interpret, as they are based on simple binary operations and geometric properties (such as eigenvalues), respectively.

The authors also consider the testing of other textural characteristics, such as Haralick or Tamura features, using other machine learning methods for the classification task.

The question of the effect of sliding window size on contour segmentation in gradient analysis methods for processing ultrasound images of the breast is also an important aspect that can significantly impact the accuracy of contour segmentation. Larger windows can capture more context but may also average out important details, while smaller windows may better reveal local characteristics, but are conversely sensitive to noise. The optimal sliding window size may vary depending on image characteristics such as resolution, noise level, and contrast. Methods such as cross-validation can be used to choose the optimal window size, where different window sizes are matched with different metrics, such as accuracy and recall. Adaptive methods that adjust the window size according to local image features may also be used.

The ultrasound beam is emitted by the transducer, whose surface corresponds to the upper border of the ultrasound image. Then, the ultrasound passes through the tissues vertically downwards and reflects in the opposite direction. These reflected echoes form the basis of the high-quality ultrasound image with complete data, which is typical of the upper lesion hemisphere. In contrast, some ultrasound may reflect from the side lesion borders; however, a part of the reflected beam in this case passes under the variable angles to the vertical, missing the transducer. In some cases, when the lesion tissue absorbs the ultrasound, it produces very little or no echo. This phenomenon corresponds to the acoustic shadow (vertical black stripe) in Figure 9b. This means that this particular lesion has no deep component data for future processing, and it is impossible to define its lower border. Therefore, limiting the segmentation to the upper hemisphere makes it possible to minimize the impact of artifacts and low-echo areas on the results of the subsequent classification of pathological lesions [4,5].

An advantage of the developed method for contouring lesions, which takes into account pixel brightness gradient characteristics, is that it does not utilize additional noise suppression methods [28,29] that could distort the lesion contours. When developing segmentation algorithms for lesions, it is crucial not to use preprocessing with spatial filters on medical images, as this may lead to distortion of the lesion’s contours. The use of such filters can also adversely affect the extraction of textural and geometric characteristics of the lesion, potentially leading to errors in their subsequent classification.

The distinguishing feature of the developed method compared to, for example, the one proposed in [6] is that the authors of this paper do not use image preprocessing methods and adaptive thresholding to determine the initial approximation point. Instead, pixel classification is performed using a random forest model. Due to the wide variability in both the sizes of the changes and their spatial positions, there is a challenge in selecting the processing window size for calculating the adaptive threshold, which can result in many false objects that are comparable in intensity to the lesions in the ultrasound images. Furthermore, the contour segmentation itself is based on the analysis of the brightness gradient of the images, avoiding area-based methods that heavily rely on the initial approximation point and may yield unstable results in cases where the background has characteristics similar to those of the examined lesion.

The proposed method was implemented in the interpretable programming language “Python v3.9” with Spyder IDE v6.0.0 on a personal computer to assess its effectiveness. For the application of this method on standard clinical ultrasound devices in real-time mode, it is necessary to implement it in more efficient, compiled programming languages (such as “C”/“C++”) and to use faster algorithms for the random forest model, such as FastForest [30], which allows for increased processing speed while maintaining accuracy.

## 5. Conclusions

The proposed method for detecting the ROI and outlining the contour of pathological lesions in breast ultrasound video sequences demonstrated an average detection rate of 91.89% per frame and an average contour extraction accuracy measured by the IoU metric of 0.871, which is negligible considering the error of manual delineation by a specialist. Thus, the proposed method can be used to highlight the ROI containing a suspicious lesion and for subsequent segmentation.

## Figures and Tables

**Table 1:** Assessment of normal tissue classification efficiency.

Tissue Type	Average Percentage of Correctly Classified Tissue Pixels
Skin	94.74%
Fat	95.14%
Glandular tissue	91.25%
Fibrous tissue	90.21%
Artifacts	86.99%

**Table 2:** Results of contour segmentation quality assessment.

Metric	Rating of the First Video	Rating of the Second Video	Mean Rating for All 52 Videos
$\bar{d}$ ± σ	13.35 ± 12.15	17.97 ± 13.99	15.37 ± 13.72
*IoU*	0.89	0.84	0.871
*TP*	0.88	0.86	0.875
*FP*	0.07	0.14	0.121
*FN*	0.12	0.14	0.134
Precision	0.92	0.86	0.91
Recall	0.88	0.86	0.88
*F*1-*score*	0.90	0.86	0.89

**Table 3:** Variability of manual ground truth segmentation.

Video Sequence	Metric: $\bar{d}$ ± σ (Pixels)
First video	12.98 ± 13.15
Second video	14.18 ± 13.90
Mean for all 52 videos	13.73 ± 13.189

**Table 4:** A comparison of the proposed method with the state of the art.

Method	Precision	Recall	*IoU*
AMS-PAN: breast ultrasound image segmentation model combining attention mechanism and multi-scale features [13]	86.28%	80.48%	81.81%
Deep weakly supervised breast tumor segmentation in ultrasound images with explicit anatomical constraints [14]	74.59%	80.41%	62.26%
Global guidance network for breast lesion segmentation in ultrasound images [15]	79.61%	81.10%	68.82%
Improved U-net MALF model for lesion segmentation in breast ultrasound images [16]	90.60%	–	86.20%
Edge-driven multi-agent reinforcement learning [17]	89.35%	98.57%	88.19%
Attention-based fusion network for breast cancer segmentation and classification [18]	82.21%	80.58%	68.60%
Proposed method	91.00%	88.00%	87.10%

**Table 5:** Results of lesion detection in ultrasound video frames.

Video Sequence	Correctly Detected Frames	Incorrectly Detected or Not Detected (Frames)
First video	53 out of 60 (88.34%)	7 out of 60 (11.66%)
Second video	54 out of 60 (90.00%)	6 out of 60 (10.00%)
Total all 52 videos	2867 out of 3120 (91.89%)	253 out of 3120 (8.11%)

## Data Availability

Data are contained within the article.
